# Vascular Deletion of HDAC6 Ameliorates Diabetic Retinal Microangiopathy and Diabetic Retinopathy in an Experimental Model of Type 1 Diabetes

**DOI:** 10.3390/cells15141244

**Published:** 2026-07-10

**Authors:** Sheila Ngumbi, Mohamed S. Gad, Mostafa Mahrous, Adil Ijaz, Marco Orecchioni, Kathryn Bollinger, Manuela Bartoli

**Affiliations:** 1 Department of Ophthalmology, Medical College of Georgia, Augusta University, Augusta, GA 30912, USA; foscash@gmail.com (S.N.); mgad@augusta.edu (M.S.G.); mmahrous@augusta.edu (M.M.); kbollinger@augusta.edu (K.B.); 2 Department of Medical Histology and Cell Biology, Faculty of Medicine, Mansoura University, Mansoura 35516, Egypt; 3 Immunology Center of Georgia, Augusta University, Augusta, GA 30912, USA; aijaz@augusta.edu (A.I.); morecchioni@augusta.edu (M.O.)

**Keywords:** diabetic retinopathy, HDAC6, retinal endothelial cells, retinal microangiopathy, retinal vascular senescence, inflammation, oxidative stress, blood-retinal barrier

## Abstract

Diabetic retinopathy (DR) is a leading cause of vision loss among working-age adults and is characterized by progressive retinal microvascular dysfunction driven by hyperglycemia-induced inflammation and oxidative stress. Histone deacetylase 6 (HDAC6), a cytoplasmic deacetylase involved in multiple cellular processes, has been implicated in vascular pathology; however, its specific role in retinal endothelial cells remains unclear. In this study, we investigated whether endothelial-specific deletion of HDAC6 protects against diabetic retinal injury. Endothelial HDAC6 knockout mice were generated using VE-cadherin–Cre recombination and subjected to streptozotocin-induced diabetes. Retinal vascular integrity, inflammatory and oxidative stress responses, endothelial senescence, and visual function were assessed during the course of hyperglycemia. Diabetic control mice developed characteristic features of DR, including vascular dysfunction, heightened inflammatory and oxidative stress responses, increased endothelial senescence, and impaired visual function. In contrast, endothelial-specific HDAC6 deletion markedly attenuated these pathological changes, preserving retinal vascular integrity and visual performance while reducing oxidative stress and inflammation. These findings identify endothelial HDAC6 as a key contributor to diabetes-induced retinal vascular injury and suggest that targeting endothelial HDAC6 may represent a promising therapeutic strategy for preventing microvascular damage and vision loss in DR.

## 1. Introduction

Diabetic retinopathy (DR) is a microvascular complication associated with both type 1 and type 2 diabetes [1]. It stands as the leading cause of vision loss among working-age adults [2]. DR is a progressive neurovascular complication characterized by microvascular abnormalities, including capillary leakage, pericyte loss, and retinal ischemia, ultimately leading to visual impairment or blindness if left untreated [3,4,5]. Despite advances in clinical management, the molecular mechanisms driving retinal microangiopathy in diabetes remain incompletely understood. Hyperglycemia-induced oxidative stress, inflammation, and epigenetic modifications contribute to endothelial cell activation and damage, thereby accelerating the progression of retinal microangiopathy [6].

Recent advances in molecular and imaging biomarkers have improved the early detection, monitoring, and risk stratification of DR. Biomarkers associated with inflammation, oxidative stress, vascular dysfunction, and neurodegeneration, combined with emerging retinal imaging technologies, are supporting more personalized approaches to DR management [7]. A better understanding of endothelial-specific mechanisms may further aid the identification of novel biomarkers and therapeutic targets for precision medicine in DR.

Histone deacetylase 6 (HDAC6), a well-studied member of the class IIb deacetylases, plays a key role in various biological processes by forming complexes with partner proteins [8,9]. Unlike other deacetylases, HDAC6 uniquely targets non-histone proteins, highlighting its broad functional range and potential as a therapeutic target for numerous diseases [10,11,12]. Emerging evidence has linked HDAC6 to vascular pathologies, including those involving inflammatory and oxidative stress pathways [13].

We previously demonstrated that HDAC6 expression is upregulated in human and experimental models of DR, as well as in human retinal endothelial cells (HRECs) exposed to high glucose. Moreover, pharmacological inhibition of HDAC6 using Tubastatin A, a selective HDAC6 inhibitor, significantly attenuated key features of DR in diabetic rats, including vascular leakage, inflammation, and oxidative stress [14]. However, because this approach reflects systemic HDAC6 inhibition, it does not identify the specific retinal cell population responsible for these protective effects. HDAC6 is broadly expressed across multiple retinal cell types, including endothelial cells, Müller glia, neurons, and microglia; therefore, pharmacological inhibition alone cannot establish endothelial-specific contributions to disease pathogenesis. To address this limitation, an endothelial cell-specific HDAC6 knockout model is required to enable direct causal assessment of endothelial HDAC6 in mediating retinal vascular dysfunction and DR progression.

By combining in vivo diabetic models with molecular and functional assessments, we aimed to determine whether loss of endothelial HDAC6 confers protection against hyperglycemia-induced retinal vascular dysfunction and to clarify its specific role in mediating the cellular mechanisms underlying DR.

## 2. Materials and Methods

### 2.1. Experimental Animals

All animal procedures conformed to the guidelines of the Association for Research in Vision and Ophthalmology (ARVO) for the humane use of animals in vision research and were approved by Augusta University-approved protocols (2009-0181) on (22 January 2024). Endothelial-specific HDAC6 knockout mice were generated by crossing HDAC6 floxed mice (C57BL/6J background; kindly provided by Dr. Tim McKinsey of University of Colorado upon permission and Material Transfer Agreement (MTA) from the originator of this strain, Dr. Patrick Mathias (Friedrich Miescher Biomedical Research Institute, Basel, CH)). We have cross-bred these mice with our transgenic mice expressing Cre recombinase under the control of the Cdh5 (VE-cadherin) promoter (B6.FVB-Tg(Cdh5^cre^)7Mlia/J; The Jackson Laboratory). Conditional knockout (HDAC6 ^Cre/flox^) and littermate control (HDAC6 ^flox^) mice were identified by PCR-based genotyping of tail DNA. All strains were confirmed to be free of retinal degeneration mutations.

Male mice (8–10 weeks old) were rendered diabetic by intraperitoneal injection of streptozotocin (55 mg/kg in 100 mM citrate buffer, pH 4.5) for five consecutive days. Hyperglycemia was confirmed two weeks post-injection (blood glucose > 250 mg/dL). Body weight was monitored weekly, and animals were maintained under sustained hyperglycemia for 12, 18, or 24 weeks prior to analysis, as illustrated in Supplementary Table S1. Both eyes were collected and analyzed separately, but statistically evaluated as one animal.

### 2.2. Visual Acuity

Visual acuity was assessed by measuring spatial frequency thresholds using a virtual optokinetic tracking system (OptoMotry; CerebralMechanics, Medicine Hat, AB, Canada) [15]. This system quantifies visual function through the optokinetic response (OKR) and optomotor response (OMR) using a two-alternative forced-choice paradigm to minimize observer bias.

Unrestrained mice were placed on a central platform surrounded by four computer monitors generating a virtual cylinder of vertically drifting sine-wave gratings (12°/s) at mean luminance. The cylinder hub was continuously centered between the animal’s eyes, and spatial frequency (SF) was adjusted automatically using a staircase algorithm. When the animal was stationary, gray screens were replaced by drifting gratings to elicit reflexive tracking responses, which were scored in real time. Spatial frequency thresholds (SFs) were determined at 100% contrast for each eye independently in an interleaved manner. Visual acuity was defined as the highest SF, eliciting tracking responses in ≥70% of trials, and is reported in cycles per degree (c/d).

### 2.3. Electroretinography (ERG)

ERG was performed to assess retinal function in vivo [16] using the Diagnosys Celeris ERG system (Lowell, MA, USA). Mice were dark-adapted overnight and anesthetized with 80 mg/kg ketamine and 10 mg/kg xylazine. Next, 2.5% Phenylephrine Hydrochloride Ophthalmic Solution and 2% Tropicamide Ophthalmic Solution were used to dilate the pupils. Probes, containing both an electrode and a light source, were placed on the cornea with Hypromellose (GenTeal^®^Tears) to prevent corneal drying. For analysis of predominantly rod function, dark adapted scotopic scans were performed. A series of light flashes with increasing intensities were presented to the eye (e.g., 0.001, 0.005, 0.01, 0.1, 0.5, 1 cd s/m^2^) and b-wave amplitudes and implicit times were measured. Following scotopic analysis, photopic scans were performed to assess cone function. Another series of light flashes with increasing intensities were presented to the eye (e.g., 3, 10, 25, 50, 100, 150 cd s/m^2^), and b-wave amplitudes and implicit times were measured and presented in units of microvolts (μV) and milliseconds (ms), respectively.

### 2.4. Assessment of Retinal Vascular Permeability

Retinal vascular permeability was evaluated by fluorescein angiography using the Phoenix Micron III retinal imaging microscope (Phoenix Research Laboratories, Pleasanton, CA, USA). Mice were anesthetized with ketamine/xylazine (80/12 mg/kg), and pupils were dilated with 1% tropicamide (Bausch & Lomb, Vaughan, ON, Canada). Goniovisc 2.5% (Hypromellose; Sigma Pharmaceuticals, North Liberty, IA, USA) was applied to maintain corneal hydration. Fluorescein sodium (10%, 20 μL; Apollo Ophthalmics, Newport Beach, CA, USA) was administered intraperitoneally, and sequential fluorescent images were captured at standardized time intervals for all experimental groups. In addition, we assessed vascular permeability quantitatively by measuring albumin extravasation to the retinal tissue as described before [17]. Briefly, following deep anesthesia, mice were perfused transcardially with phosphate-buffered saline via left ventricular cannulation, with drainage through the right atrium, to remove intravascular blood. Retinas were harvested, and albumin accumulation in perfused retinal tissue was determined by Western blot analysis using an anti-albumin antibody (Cell Signaling Technology, Danvers, MA, USA).

### 2.5. Analysis of Leukocyte Adhesion

Leukocyte adhesion to the retinal vasculature was assessed using a fluorescein isothiocyanate (FITC)–concanavalin A (Con A) lectin perfusion assay as previously described [18]. Following deep anesthesia (ketamine/xylazine, 80/12 mg/kg), the thoracic cavity was opened, and a 20-gauge cannula was inserted into the aorta, with drainage established via incision of the right atrium. Mice were perfused with phosphate-buffered saline (PBS) to remove non-adherent blood cells, followed by perfusion with FITC-labeled Con A (40 μg/mL in PBS; Vector Laboratories, Newark, CA, USA) to label adherent leukocytes and vascular endothelial cells. Excess lectin was cleared by additional PBS perfusion. Eyes were enucleated and fixed in 4% paraformaldehyde. Retinas were flat-mounted and imaged using a Nikon AX confocal microscope (Nikon, Melville, NY, USA). The total number of adherent leukocytes per retina was quantified. CD45 immunohistochemistry was performed using an anti-CD45 antibody (BD PharMingen, San Jose, CA, USA) to confirm the leukocyte identity of Con A-positive intravascular cells.

### 2.6. Flow Cytometry

Freshly harvested retinas were dissected, and pooled samples (2–3 retinas per group) were incubated in a digestion buffer composed of RPMI 1640 medium supplemented with 0.1% collagenase type II and 10 μg/mL DNase I. The tissue was finely minced and passed through a 70 μm nylon mesh to generate a single-cell suspension, followed by incubation at 37 °C for 20–30 min. Cells were gently dispersed by pipetting and subsequently filtered through a 40 μm cell strainer. The resulting suspension was centrifuged at 1500 rpm for 10 min. After discarding the supernatant, the cell pellet was washed twice with phosphate-buffered saline (PBS) containing 2% fetal bovine serum (FBS).

For immunostaining, cells were incubated for 20 min at room temperature in the dark with a panel of conjugated antibodies in Table 1, including Alexa Fluor 700-conjugated anti-CD45, PE/Cy7-conjugated anti-mouse/human CD11b, FITC-conjugated anti-Ly6G, Live/Dead Blue viability dye, APC/Cy7-conjugated anti-CD19, and APC-eFluor 780-conjugated anti-TCRβ, which were used as a dump channel.

Following staining, cells were washed and fixed with 2% paraformaldehyde (PFA) for 15 min at room temperature in the dark. After a final wash, samples were analyzed using a Cytek 5-Laser Aurora Spectral Cytometer (Cytek Biosciences, Inc., Fremont, CA, USA). Data was processed and analyzed with FlowJo 10 software (BD Biosciences, San Jose, CA, USA).

### 2.7. Flat Mounts

Mice were perfused transcardially with phosphate-buffered saline (PBS, pH 7.4), and eyes were immediately enucleated and fixed in 4% paraformaldehyde for 4 h at room temperature. Following fixation, tissues were cryoprotected in 30% sucrose overnight. Retinas were carefully dissected, washed in PBS containing 0.1% Triton X-100 (PBS-T), and permeabilized in 3% Triton X-100 for 1 h.

Samples were blocked in 5% normal goat serum prepared in PBS-T and incubated with primary antibodies against BRN3A (Synaptic Systems, Goettingen, Germany) or RBPMS (Antibodies Incorporate, Davis, CA, USA) at 4 °C for 48 h. After extensive washing, retinas were incubated overnight at 4 °C with appropriate Alexa Fluor–conjugated goat anti-rabbit secondary antibodies. Following final washes, retinas were flat-mounted with antifade mounting medium.

Imaging was performed using a Nikon AX confocal microscope equipped with the NSPARC imaging system (Nikon, Melville, NY, USA).

### 2.8. Dot Blot Analysis

Equivalent amounts of proteins prepared from whole mouse retina lysates were spotted on nitrocellulose membranes and dried for 5 min at room temperature. The membranes were blocked for 1 h by using 5% BSA in TBS-T and then probed for either anti-3-nitrotyrosine (3-NT 1:1000; Bioss Inc., Woburn, MA, USA) or anti 4-hydroxynonenal (4-HNE,1:1000; Bioss Inc., Woburn, MA, USA) antibodies overnight at 4 °C. The membranes were then washed three times and probed again with horseradish peroxidase-conjugate secondary antibody (Cell Signaling Technology, Danvers, MA, USA). After washing the membrane, the immuno-positive spots were visualized by using Clarity ECL-Blotting substrate (Bio-Rad, Hercules, CA, USA). Scanned images of blots were used to quantify protein expression using NIH ImageJ software (Version 1.54).

### 2.9. Immunohistochemical Analysis

Mouse eyes were embedded in OCT mounting medium (Tissue-Tek, Torrance, CA, USA), frozen on dry ice and then cryostat-sectioned. A 4% paraformaldehyde fixative was applied to the slides for 10 min. Slides were incubated overnight at 4 °C with one of the following primary antibodies: rabbit anti- myeloperoxidase (MPO) (Abcam, Cambridge, MA, USA) and mouse anti-phosphorylated form of H2A histone family member X (γH2AX) (Cell Signaling Technology, Danvers, MA, USA). Slides were washed three times with 0.1% Triton X-100 in 0.1 M PBS (pH 7.4) followed by incubation with appropriate fluorescence-conjugated secondary antibodies, all purchased from Molecular Probes-Life Technologies (Grand Island, NY, USA): goat anti-rabbit IgG-conjugated Alexa Fluor 488, goat anti-mouse IgG-conjugated Alexa Fluor 488. Slides were mounted using Fluoroshield mounting medium containing 4′,6-diamidino-2-phenylindole (DAPI) to visualize nuclei (Sigma-Aldrich, Burlington, MA, USA). Images were captured at 20X magnification using Nikon AX confocal with an NSPARC microscope.

### 2.10. Protein Analysis

Retinal proteins were extracted using RIPA lysis buffer (Thermo Fisher Scientific, Florence, SC, USA) supplemented with 1% protease and phosphatase inhibitor cocktail. Protein concentrations were determined using a Coomassie-based assay according to the manufacturer’s instructions. Equal amounts of protein were separated by SDS–PAGE and transferred onto polyvinylidene difluoride membranes.

Membranes were incubated overnight at 4 °C with primary antibodies against p21^Waf/Cip1^ (Santa Cruz Biotechnology, Dallas, TX, USA), albumin and β-actin (Cell Signaling Technology, MA, USA), and ICAM-1 (Abclonal, Woburn, MA, USA). After washing with TBST, blots were incubated with horseradish peroxidase-conjugated secondary antibodies (1:3000; Sigma-Aldrich, MA, USA) for 1 h at room temperature. Immunoreactive bands were visualized using Clarity ECL substrate (Bio-Rad, CA, USA). β-actin served as a loading control. Band intensities were quantified from scanned images using NIH ImageJ software.

### 2.11. Cytokine Assay

To determine the levels of inflammatory cytokines in retinal lysate, we used a customized mouse Mix and Match Cytokine ELISA strip assay (Signosis, Santa Clara, CA, USA). Retinal tissue samples from different experimental groups were homogenized using 1x cell lysis buffer (Signosis, Santa Clara, CA, USA), and total protein concentration was quantified using the Coomassie Plus (Bradford) Assay Kit (Thermo Fisher Scientific, Waltham, MA, USA). Samples containing an equivalent amount of proteins were added to different wells individually coated with primary antibodies against TNFα (tumor necrosis factor α), IL-10, IL-1β, and IL-6. Plates were processed according to the manufacturer’s instructions and were read at 450 nm. Protein standards provided by the manufacturer were used to calculate the concentration of each cytokine, which was expressed as ng/mg of total protein content.

### 2.12. Statistical Analysis

All data sets were expressed as mean ± SEM and analyzed using GraphPad Prism 10 (GraphPad 10 Software Inc., La Jolla, CA, USA). Multiple comparisons were performed using Two-way ANOVA with the Tukey post hoc test. For all comparisons, *p* values < 0.05 were considered significant. Statistical units indicated in text refer to the number of animals; however, each data point is the average of both eyes/per animal (when *n* = 4, *n* = 8 eyes).

## 3. Results

### 3.1. Generation and Validation of Endothelial-Specific HDAC6 Knockout Mice

**Figure 1:**
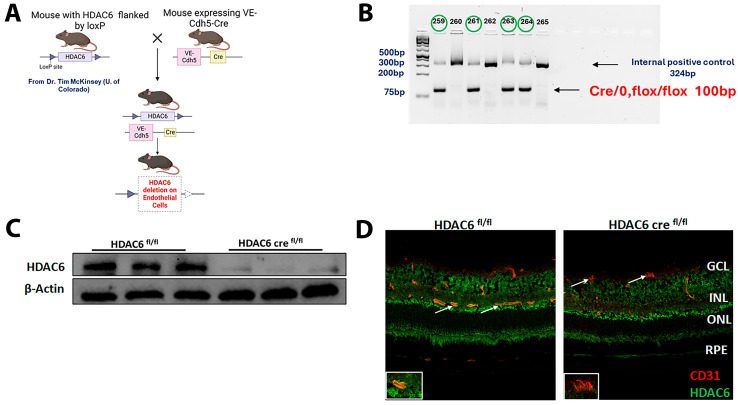
Generation and validation of endothelial-specific HDAC6 knockout mice. (**A**) Breeding strategy to generate endothelial-specific HDAC6 knockout mice by crossing HDAC6 ^fl/fl^ mice with VE-cadherin (Cdh5)-Cre mice. (**B**) PCR genotyping confirming the HDAC6 cre ^fl/fl^ genotype. (**C**) Western blot showing reduced HDAC6 protein levels in retinas from HDAC6 cre ^fl/fl^ mice compared with HDAC6 ^fl/fl^ controls. (**D**) Immunofluorescence co-staining for HDAC6 and CD31 demonstrating loss of HDAC6 in retinal endothelial cells, with preserved expression in non-endothelial cells.

To investigate the endothelial-specific contribution of HDAC6, we crossed HDAC6 ^fl/fl^ mice with VE-Cadherin 5-Cre mice to generate HDAC6 cre ^fl/fl^ offspring (Figure 1A). Cre-mediated recombination results in excision of the floxed HDAC6 allele specifically in endothelial cells. The desired genotype was obtained at the third generation, as confirmed by PCR genotyping of tail DNA (Figure 1B). To assess the efficiency of HDAC6 deletion, retinal protein extracts from HDAC6 cre ^fl/fl^ and HDAC6 ^fl/fl^ were analyzed by Western blot. A pronounced decrease in HDAC6 protein levels was detected in HDAC6 cre ^fl/fl^ retinas (Figure 1C). In addition, immunofluorescence co-staining for HDAC6 and the endothelial marker CD31 revealed marked loss of HDAC6 expression in retinal endothelial cells, whereas non-endothelial cells preserved HDAC6 signal (Figure 1D). Collectively, these results confirmed efficient and endothelial-specific deletion of HDAC6, establishing HDAC6 cre ^fl/fl^ mice as a reliable model for downstream functional studies.

### 3.2. Endothelial HDAC6 Contributes to Hyperglycemia-Induced Retinal Vascular Senescence

**Figure 2:**
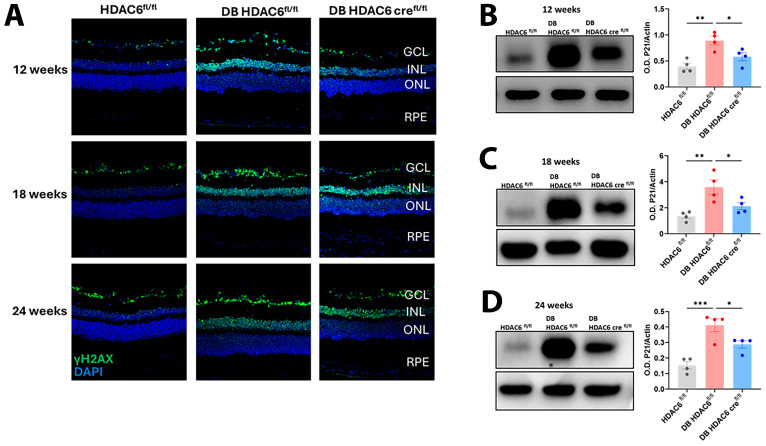
Endothelial HDAC6 promotes hyperglycemia-induced retinal vascular senescence. (**A**) Representative retinal immunohistochemistry for the senescence marker γH2AX showing increased immunoreactivity in diabetic HDAC6 ^fl/fl^ mice, particularly in the ganglion cell layer (GCL) and inner nuclear layer (INL), compared with non-diabetic controls. This increase is attenuated in diabetic endothelial-specific HDAC6 cre ^fl/fl^ mice. (**B**–**D**) Western blot analysis and quantification of the senescence-associated protein p21 in retinal extracts demonstrate elevated p21 levels in diabetic HDAC6 ^fl/fl^ mice, which are significantly reduced by endothelial-specific deletion of HDAC6 across all examined time points. (* *p* <0.05, ** *p* < 0.01, *** *p* < 0.001; *n* = 4).

Cellular senescence is increasingly recognized as a contributor to vascular dysfunction in DR, where chronic hyperglycemia accelerates stress-induced damage and impairs tissue repair [19]. To investigate the role of endothelial HDAC6 in retinal vascular senescence during diabetes, we examined senescence markers in retinal tissue from control, diabetic HDAC6 ^fl/fl^ and endothelial-specific HDAC6 cre ^fl/fl^ mice. Immunohistochemical analysis of the senescence marker, the phosphorylated form of H2A histone family member X (γH2AX), showed increased immunoreactivity in diabetic HDAC6 ^fl/fl^ mice, particularly within the ganglion cell layer (GCL) and inner nuclear layer (INL), compared to the non-diabetic control (Figure 2A). However, in diabetic HDAC6 cre ^fl/fl^ mice, this elevation was consistently attenuated (Figure 2A).

To corroborate these findings, we assessed protein levels of p21, a cyclin-dependent kinase inhibitor and key regulator of senescence [20,21]. Western blot analysis revealed increased p21 protein levels in the retinas of diabetic HDAC6 ^fl/fl^ mice compared to control HDAC6 ^fl/fl^ (Figure 2B–D). Importantly, endothelial-specific deletion of HDAC6 significantly reduced p21 expression across all time points, reinforcing the role of HDAC6 in driving vascular senescence under diabetic conditions (Figure 2B–D).

### 3.3. Endothelial HDAC6 Deletion Preserves Blood–Retinal Barrier Integrity in Diabetic Retinopathy

**Figure 3:**
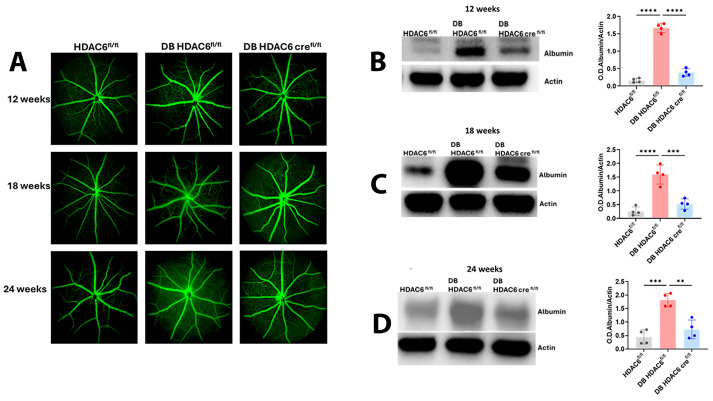
Endothelial HDAC6 deletion preserves blood–retinal barrier integrity in diabetic retinopathy. (**A**) Representative fluorescein angiography images showing a time-dependent increase in vascular leakage in diabetic HDAC6 ^fl/fl^ mice at 12, 18, and 24 weeks post-diabetes induction, with maximal hyperfluorescence at 24 weeks. This leakage is markedly reduced in diabetic endothelial-specific HDAC6 cre ^fl/fl^ mice. (**B**–**D**) Western blot analysis and quantification of retinal albumin demonstrating increased albumin extravasation in diabetic HDAC6 ^fl/fl^ mice, indicative of blood–retinal barrier breakdown, which is significantly attenuated by endothelial-specific deletion of HDAC6. ** *p* < 0.01, *** *p* < 0.001 or **** *p* < 0.0001, *n* = 4).

Disruption of the blood–retinal barrier (BRB) and subsequent vascular leakage are hallmark features of DR [22,23]. To elucidate the role of endothelial HDAC6 in hyperglycemia-induced vascular permeability, we examined control and endothelial HDAC6 knockout mice at 12, 18, and 24 weeks post-diabetes induction. Vascular leakage was evaluated qualitatively via fluorescein angiography and quantitatively by assessing retinal albumin extravasation through Western blot analysis. Fluorescein angiography revealed a time-dependent increase in hyperfluorescent regions in diabetic HDAC6 ^fl/fl^ mice relative to non-diabetic controls, with maximal leakage observed at 24 weeks (Figure 3A). In contrast, diabetic HDAC6 cre ^fl/fl^ mice, harboring endothelial-specific HDAC6 deletion, exhibited significantly reduced hyperfluorescence across all time points, indicating a protective effect against diabetes-induced vascular compromise (Figure 3A). Consistent with these findings, Western blot analysis demonstrated elevated retinal albumin levels in diabetic HDAC6 ^fl/fl^ mice, indicative of BRB breakdown (Figure 3B–D). This increase was substantially mitigated in HDAC6 cre ^fl/fl^ counterparts, reinforcing the role of endothelial HDAC6 in mediating vascular leakage under hyperglycemic conditions. Collectively, these data implicate endothelial HDAC6 as a critical contributor to BRB dysfunction in diabetic retinopathy and suggest that its targeted deletion confers vascular protection throughout the progression of diabetes.

### 3.4. Lack of Endothelial HDAC6 Diminishes Hyperglycemia-Induced Retinal Inflammation

Chronic inflammation is a pivotal contributor to the pathogenesis of DR, exacerbating vascular dysfunction and tissue damage in the retina [24,25]. To investigate the contribution of endothelial HDAC6 to hyperglycemia-induced retinal inflammation, we assessed leukocyte adhesion in the retinal vasculature of diabetic HDAC6 ^fl/fl^ mice and mice lacking the expression of HDAC6 in endothelial cells using concanavalin A labeling [26].

**Figure 4:**
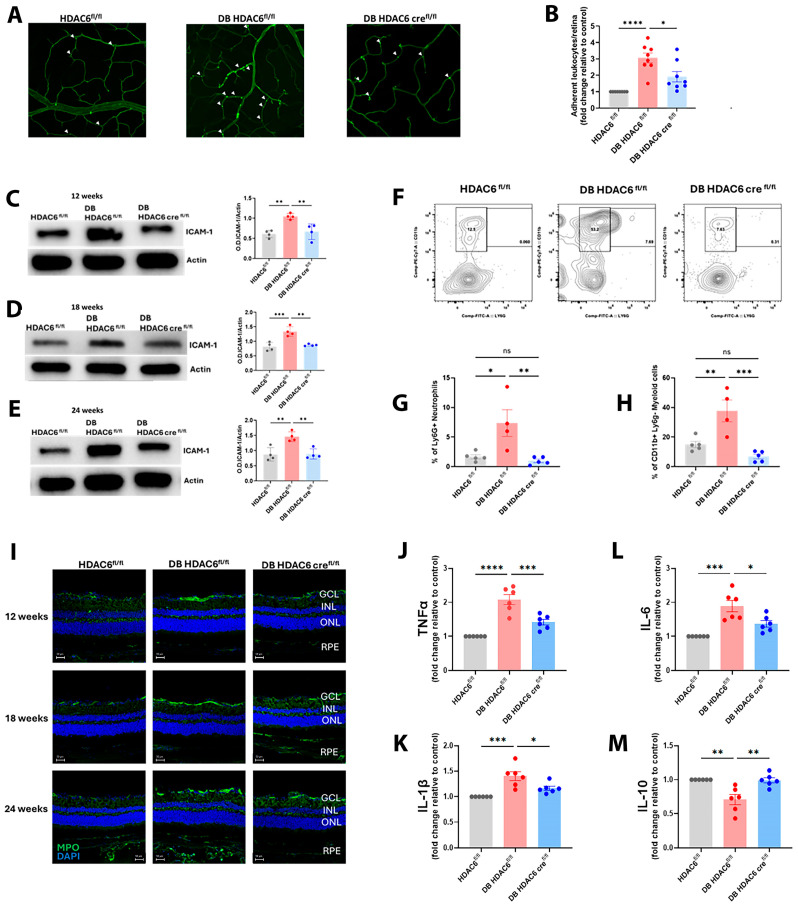
Endothelial HDAC6 deletion attenuates hyperglycemia-induced retinal inflammation. (**A**,**B**) Representative concanavalin A labeling and quantification showing increased leukocyte adhesion in the retinal vasculature of diabetic HDAC6 ^fl/fl^ mice compared with non-diabetic controls, which is markedly reduced in diabetic endothelial-specific HDAC6 cre ^fl/fl^ mice. (*n* = 8) (**C**–**E**) Western blot analysis and quantification demonstrating upregulation of ICAM-1 in retinas from diabetic HDAC6 ^fl/fl^ mice, with significant reduction following endothelial HDAC6 deletion. (*n* = 4) (**F**–**H**) Flow cytometric analysis revealing increased accumulation of neutrophils (CD11b^+^ Ly6G^+^) and myeloid cells (CD11b^+^ Ly6G^−^) in diabetic HDAC6 ^fl/fl^ retinas, which is significantly attenuated in HDAC6 cre ^fl/fl^ mice. (*n* = 5), (**I**) Immunofluorescence staining for myeloperoxidase (MPO) showing elevated neutrophil activation in diabetic HDAC6 ^fl/fl^ retinas, reduced by endothelial-specific HDAC6 deletion. (**J**–**M**) Retinal cytokine profiling demonstrating increased pro-inflammatory cytokines (IL-1β, IL-6, TNF-α) and reduced anti-inflammatory IL-10 in diabetic HDAC6 ^fl/fl^ mice; these alterations are normalized in HDAC6 cre ^fl/fl^ mice. Collectively, these data identify endothelial HDAC6 as a key driver of retinal inflammation in diabetic retinopathy. (*n* = 6) (ns = non significant, * *p* < 0.05, ** *p* < 0.01, *** *p* < 0.001 or **** *p* < 0.0001).

Analysis of leukocyte adhesion showed a significant increase in the number of adherent leukocytes in the retinas of diabetic HDAC6 ^fl/fl^ mice compared to control HDAC6 ^fl/fl^ mice (Figure 4A,B). Notably, endothelial-specific deletion of HDAC6 markedly reduced leukocyte adhesion in retinas of diabetic HDAC6 cre ^fl/fl^ mice, indicating a significant reduction in this inflammatory response (Figure 4A,B).

This was paralleled by changes in the retinal expression of intercellular adhesion molecule-1 (ICAM-1), a key mediator of leukocyte–endothelial interactions [27,28,29] which was upregulated in the retinas of diabetic HDAC6 ^fl/fl^ mice but significantly reduced in diabetic HDAC6 cre ^fl/fl^ mice lacking endothelial HDAC6 (Figure 4C–E).

To further characterize the inflammatory cell populations recruited to the retina, we performed flow cytometric analysis of retinal slurry to identify the types of cells trapped within the retinal tissue. Our data revealed that neutrophils, identified as CD11b^+^Ly6G^+^ cells, and myeloid cells, identified as CD11b^+^Ly6G^−^ cells, were significantly increased in the retinas of diabetic HDAC6 ^fl/fl^ mice compared to normoglycemic HDAC6 ^fl/fl^ controls (Figure 4F–H). Interestingly, deletion of HDAC6 in endothelial cells markedly reduced the accumulation of both neutrophils and myeloid cells in the diabetic retina, suggesting that endothelial HDAC6 plays a crucial role in mediating immune cell infiltration under hyperglycemic conditions (Figure 4F–H).

Additionally, immunofluorescence staining for myeloperoxidase (MPO), a marker of neutrophil activation and oxidative stress [30], revealed elevated MPO expression in the retinas of diabetic HDAC6 ^fl/fl^ mice compared to non-diabetic controls (Figure 4I). This increase was significantly reduced in diabetic HDAC6 cre ^fl/fl^ mice at all time points analyzed, further supporting a key role for endothelial HDAC6 in promoting inflammatory cell activation and oxidative damage in the diabetic retina.

Further analysis of retinal cytokine profiles using a custom cytokine ELISA array revealed elevated levels of pro-inflammatory cytokines IL-1β, IL-6, and TNF-α in diabetic HDAC6 ^fl/fl^ mice (Figure 4J–M). These increases were consistently abrogated in HDAC6 cre ^fl/fl^ mice, underscoring the role of endothelial HDAC6 in amplifying retinal inflammation. Conversely, the anti-inflammatory cytokine IL-10 was significantly reduced in diabetic HDAC6 ^fl/fl^ mice, whereas endothelial HDAC6 deletion restored IL-10 levels to those observed in non-diabetic controls (Figure 4J–M).

Together, these findings identify endothelial HDAC6 as a central regulator of inflammation in our experimental model of DR. By modulating the expression of adhesion molecules, immune cell trafficking, and cytokine balance, HDAC6 contributes to the persistent inflammatory state that exacerbates retinal vascular dysfunction. Targeted inhibition or deletion of endothelial HDAC6 may therefore represent a promising therapeutic strategy for suppressing inflammation-driven retinal injury and preserving vascular integrity in diabetes.

### 3.5. Endothelial HDAC6 Knockdown Diminishes Hyperglycemia-Induced Oxidative/Nitrative Stress

Oxidative stress is a key pathological feature of DR, contributing to endothelial dysfunction, inflammation, and retinal neurovascular damage [19,31]. To assess the role of endothelial HDAC6 in mediating oxidative stress under hyperglycemic conditions, we quantified the formation of two well-established oxidative stress markers: 3-nitrotyrosine (3-NT), indicative of peroxynitrite formation, and 4-hydroxynonenal (4-HNE), a reactive lipid peroxidation product in retinal extracts from diabetic HDAC6 ^fl/fl^ and HDAC6 cre ^fl/fl^ mice using dot blot analysis.

**Figure 5:**
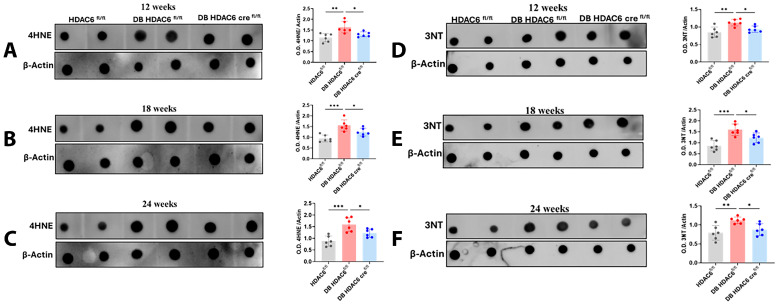
Endothelial HDAC6 knockdown attenuates hyperglycemia-induced oxidative and nitrative stress. Dot blot analysis and quantification showing increased retinal levels of 4-hydroxynonenal (4-HNE) (**A**–**C**) and 3-nitrotyrosine (3-NT) (**D**–**F**) in diabetic HDAC6 ^fl/fl^ mice at all examined time points, indicating enhanced oxidative and nitrative stress. Meanwhile, endothelial-specific deletion of HDAC6 significantly reduces retinal 3-NT and 4-HNE levels in diabetic HDAC6 cre ^fl/fl^ mice across all time points. These data demonstrate that endothelial HDAC6 contributes to hyperglycemia-induced oxidative damage in the retina. (* *p* < 0.05, ** *p* < 0.01, or *** *p* < 0.001, *n* = 6).

As demonstrated in (Figure 5A–C), diabetes markedly increased the levels of both 3-NT and 4-HNE in the retinas of HDAC6 ^fl/fl^ mice at all time points, reflecting enhanced oxidative damage in response to chronic hyperglycemia. In contrast, mice lacking HDAC6 specifically in endothelial cells exhibited a marked reduction in both 3-NT and 4-HNE levels across all time points (Figure 5D–F).

These findings support the conclusion that endothelial HDAC6 contributes to the generation of reactive nitrogen and oxygen species in response to hyperglycemia, further implicating it in the oxidative damage associated with diabetic retinopathy.

### 3.6. Deletion of Endothelial HDAC6 Reduces the Degeneration of Retinal Ganglion Cells and Prevents Diabetes-Induced Retinal Cell Death

**Figure 6:**
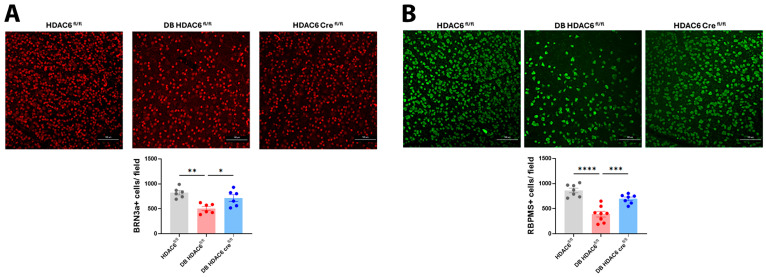
Endothelial HDAC6 deletion preserves retinal ganglion cells and reduces diabetes-induced retinal cell death. (**A**,**B**) Representative retinal flat mounts and quantification of retinal ganglion cell (RGC) density identified by Brn3a (**A**) and RBPMS (**B**) immunostaining. Diabetic HDAC6 ^fl/fl^ mice exhibit a significant reduction in Brn3a ^+^ and RBPMS^+^ RGC density compared with non-diabetic controls, whereas endothelial-specific HDAC6 cre ^fl/fl^ mice show preserved RGC densities under diabetic conditions. These data indicate that deletion of endothelial HDAC6 protects against diabetes-induced retinal neurodegeneration. (* *p* < 0.05, ** *p* < 0.01, *** *p* < 0.001 or **** *p* < 0.0001, *n* = 6–8).

Neurodegeneration of the retina is a key event in diabetic retinopathy, and significant loss of retinal ganglion cells (RGCs) has been consistently reported in association with diabetes [32,33]. To evaluate whether endothelial HDAC6 contributes to this neurodegenerative process, we quantitatively analyzed RGCs in retinal flat mounts from HDAC6 ^fl/fl^ and HDAC6 cre ^fl/fl^ mice under normoglycemic and diabetic conditions. RGCs were identified using Brn3a and RBPMS immunostaining and expressed as cell density (cells/mm^2^). As expected, diabetic HDAC6 ^fl/fl^ mice exhibited a marked reduction in Brn3a^+^ (Figure 6A) and RBPMS^+^ (Figure 6B) cell density compared with non-diabetic HDAC6 ^fl/fl^, confirming diabetes-induced RGC loss. In contrast, diabetic HDAC6 cre ^fl/fl^ mice showed significantly higher RGC densities, closely resembling those of non-diabetic HDAC6 ^fl/fl^ mice (Figure 6A,B). Together, these findings indicate that endothelial HDAC6 deletion mitigates diabetes-induced retinal neurodegeneration by preserving RGC survival.

### 3.7. Knockdown of Endothelial HDAC6 Preserves Visual Function in Diabetic Mice

To assess whether knockdown of endothelial HDAC6 affects visual function, we employed a visual acuity test. Using a virtual optokinetic system (OptoMotry, CerebralMechanics, Medicine Hat, AB, Canada) [15], we measured optomotor reflex-based spatial frequency thresholds as an index of visual acuity.

**Figure 7:**
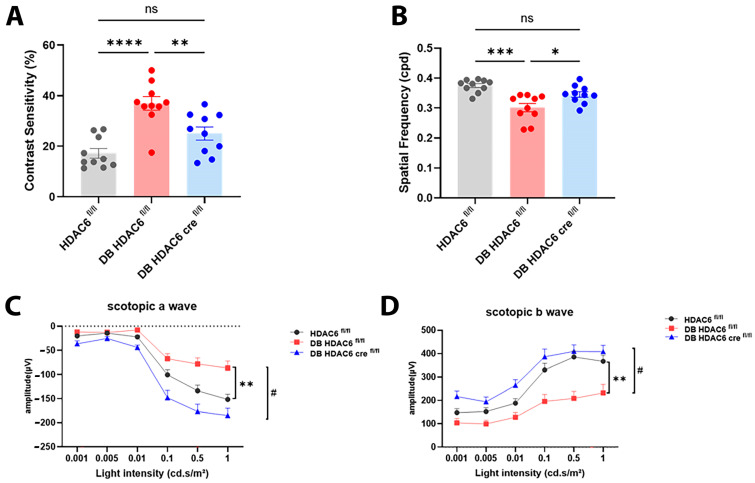
Endothelial HDAC6 deletion preserves visual and retinal function in diabetic mice. (**A**,**B**) Optomotor response analysis showing reduced contrast sensitivity (**A**) and spatial frequency thresholds (**B**) in diabetic HDAC6 ^fl/fl^ mice compared with non-diabetic controls. Endothelial-specific deletion of HDAC6 significantly improves both parameters in diabetic HDAC6 cre ^fl/fl^ mice. (**C**,**D**) Electroretinography (ERG) recordings demonstrating decreased scotopic a-wave (**C**) and b-wave (**D**) amplitudes in diabetic HDAC6 ^fl/fl^ mice, indicative of impaired photoreceptor and inner retinal function. These deficits are markedly attenuated in diabetic HDAC6 cre ^fl/fl^ mice, with responses comparable to non-diabetic controls. Collectively, these data indicate that endothelial HDAC6 contributes to diabetes-induced visual dysfunction and that its deletion preserves retinal function. (* *p* < 0.05, ** *p* < 0.01, *** *p* < 0.001 or **** *p* < 0.0001, ns: non significant, #; highlu significant *n* = 10).

Diabetic HDAC6 ^fl/fl^ mice exhibited a significant reduction in both contrast sensitivity (Figure 7A) and spatial frequency thresholds (Figure 7B) compared to non-diabetic controls, indicating impaired visual function. Interestingly, endothelial HDAC6 deletion restored visual acuity in diabetic HDAC6 cre ^fl/fl^ mice, as evidenced by the significant improvement in visual acuity and contrast sensitivity compared to diabetic HDAC6 ^fl/fl^ mice (Figure 7A,B).

To further evaluate retinal function, we performed electroretinography (ERG). As expected, diabetic HDAC6 ^fl/fl^ mice showed diminished scotopic a-wave (Figure 7C) and b-wave (Figure 7D) amplitudes, consistent with compromised photoreceptor function and impaired inner retina function, respectively. In contrast, diabetic HDAC6 cre ^fl/fl^ mice showed significantly improved ERG responses across all parameters, with amplitudes comparable to those of non-diabetic controls.

Taken as a whole, these results suggest that endothelial HDAC6 plays a crucial role in mediating visual and retinal impairments associated with diabetes. Inactivation of HDAC6 in endothelial cells helps to preserve retinal structure and function, underscoring its promise as a therapeutic target for rescuing overall visual function in DR.

## 4. Discussion

DR is a complex neurovascular disease driven by chronic hyperglycemia-induced endothelial dysfunction, inflammation, oxidative stress, and progressive neurodegeneration [34,35,36,37]. Although epigenetic regulators have emerged as important contributors to diabetic complications, their cell type-specific roles in DR remain incompletely defined. Our previous study demonstrated that systemic pharmacological inhibition of HDAC6 with Tubastatin A attenuates retinal inflammation, oxidative stress, and vascular dysfunction in the diabetic retina [14] however, such systemic inhibition affects multiple retinal and non-retinal cell types, making it difficult to delineate whether the observed protection is mediated directly through endothelial HDAC6 or indirectly via other cellular populations.

In the present study, we provide genetic evidence that, in our experimental model of DR, endothelial HDAC6 is a central driver of diabetic retinal microangiopathy and neurovascular dysfunction. Using an endothelial-specific HDAC6 knockout mouse model, we demonstrated that loss of endothelial HDAC6 markedly attenuates hyperglycemia-induced vascular senescence, preserves blood–retinal barrier (BRB) integrity, suppresses inflammation and oxidative/nitrative stress, protects retinal ganglion cells (RGCs), and ultimately preserves visual function.

A key finding of this study is the identification of endothelial HDAC6 as a regulator of hyperglycemia-induced vascular senescence in the retina. Cellular senescence is gaining increasing recognition as a pathogenic mechanism in DR [38,39], contributing to endothelial dysfunction, impaired vascular repair, and chronic inflammation. Diabetic HDAC6 ^fl/fl^ mice exhibited robust increases in γH2AX immunoreactivity and p21 expression, indicating enhanced DNA damage and cell cycle arrest. Importantly, endothelial-specific deletion of HDAC6 significantly blunted these senescence markers across all examined time points. These findings suggest that HDAC6 promotes stress-induced senescence in retinal endothelial cells under hyperglycemic conditions, likely exacerbating vascular dysfunction. Given that senescent endothelial cells adopt a pro-inflammatory and pro-oxidant phenotype, the observed reduction in senescence may represent a critical upstream mechanism underlying the vascular protection seen in endothelial-specific HDAC6-deficient mice.

Disruption of the BRB is a hallmark of DR and a major cause of vision-threatening complications such as diabetic macular edema [40]. Our data demonstrate that endothelial HDAC6 deletion confers robust protection against diabetes-induced BRB breakdown. Diabetic HDAC6 ^fl/fl^ mice showed progressive vascular leakage, as evidenced by fluorescein angiography and increased retinal albumin extravasation, whereas HDAC6 cre ^fl/fl^ mice exhibited significantly reduced leakage at all disease stages. These findings indicate that endothelial HDAC6 is required for hyperglycemia-induced increases in vascular permeability. Mechanistically, HDAC6 has been shown to regulate cytoskeletal dynamics, microtubule acetylation, and endothelial junctional stability [41,42]. Thus, its deletion may preserve endothelial barrier integrity by stabilizing cell–cell junctions and limiting cytoskeletal rearrangements that promote leakage in diabetes.

Inflammation is another central component of DR pathogenesis [43,44], and our results identify endothelial HDAC6 as a key amplifier of retinal inflammatory responses. Diabetic HDAC6 ^fl/fl^ mice exhibited increased leukocyte adhesion to the retinal vasculature that correlated with increased ICAM-1 expression. This in turn augmented the recruitment and adhesiveness of neutrophils and myeloid cells, leading to enhanced MPO immunoreactivity. These inflammatory changes were consistently and significantly reduced in mice lacking endothelial HDAC6. Furthermore, cytokine profiling revealed that endothelial HDAC6 deletion normalized the diabetic imbalance between pro-inflammatory cytokines (IL-1β, IL-6, TNF-α) and the anti-inflammatory cytokine IL-10. Collectively, these data suggest that endothelial HDAC6 orchestrates leukocyte–endothelial interactions and inflammatory signaling in the diabetic retina, likely contributing to sustained vascular injury and tissue and functional damage.

Oxidative and nitrative stress are closely intertwined with inflammation and vascular dysfunction in DR [45,46]. We observed marked increases in retinal 3-nitrotyrosine and 4-hydroxynonenal levels in diabetic HDAC6 ^fl/fl^ mice, reflecting enhanced peroxynitrite formation and lipid peroxidation. Endothelial-specific HDAC6 deletion significantly attenuated both markers, indicating reduced oxidative and nitrative stress. These findings are consistent with prior reports linking HDAC6 activity to reactive oxygen and nitrogen species generation [14,47] and suggest that endothelial HDAC6 acts upstream of oxidative damage in the diabetic retina. Reduced oxidative stress likely contributes to the observed improvements in endothelial function, decreased inflammation, and neuronal survival.

Importantly, our study extends beyond vascular endpoints to demonstrate that endothelial HDAC6 critically influences retinal neurodegeneration and visual function. Diabetic HDAC6 ^fl/fl^ mice showed significant loss of Brn3a^+^ and RBPMS^+^ RGCs, consistent with previous reports of early neuronal loss in DR [48]. In contrast, endothelial HDAC6 deletion preserved RGC density under diabetic conditions, indicating preservation of neuronal structure against diabetes-induced neuronal cell death. These structural benefits translated into functional improvements, as HDAC6 cre ^fl/fl^ mice exhibited restored contrast sensitivity, spatial frequency thresholds, and ERG a- and b-wave amplitudes. These findings support the concept that vascular health and neuronal survival are tightly coupled in DR and suggest that targeting endothelial HDAC6 can preserve retinal neurovascular integrity and visual function.

As limitation of our studies, we acknowledge that our experimental model accounts for pathophysiological changes associated with DR in Type 1 diabetes (T1D) and cannot be completely extended to Type 2 diabetic (T2D) models. However, DR incidence in T1D largely exceeds that in T2D (70–80% versus 25–34% respectively) [49], increasing the potential translational significance of our findings. Nevertheless, because of the much larger number of Type 2 diabetic patients, the validation of HDAC6 inhibition as a potential therapeutic strategy in DR in T2D should be further addressed in future work.

## 5. Conclusions

In conclusion, this study identifies endothelial HDAC6 as a central epigenetic regulator of diabetic retinal microangiopathy, inflammation, oxidative stress, neurodegeneration, and visual dysfunction. Genetic deletion of endothelial HDAC6 confers sustained vascular and neuronal protection throughout diabetes progression. These findings highlight endothelial HDAC6 as a promising therapeutic target and support the development of HDAC6-directed strategies aimed at preserving retinal structure and function in DR.

## Figures and Tables

**Table 1:** Antibodies used for flow cytometry.

Antibody	Clone	Dilution	Catalogue	Manufacturer
Anti-mouse CD45-AF700	30-F11	1:400	103127	BioLegend, San Diego, CA, USA
Anti-mouse/human CD11b-PE/CY7	M1/70	1:200	101216	BioLegend, CA, USA
Anti-mouse Ly6G-FITC	1A8	1:100	127606	BioLegend, CA, USA
Anti-mouse CD19-APC/CY7	6D5	1:200	115530	BioLegend, CA, USA
Anti-mouse TCRβ-APC eflour780	H57-597	1:200	47-5961-82	Invitrogen, Carlsbad, CA, USA
Live/Dead Fixable Blue Dead Cell stain	N/A	1:1000	L23105	Invitrogen, CA, USA

## Data Availability

The original contributions presented in this study are included in the article/Supplementary Materials. Further inquiries can be directed to the corresponding author.

## Supplementary Materials

The following supporting information can be downloaded at https://www.mdpi.com/article/10.3390/cells15141244/s1.

## Abbreviations

The following abbreviations are used in this manuscript:

DR	Diabetic retinopathy
HDAC6	Histone deacetylase 6
ARVO	Association for Research in Vision and Ophthalmology
Cdh5	VE-cadherin
PCR	Polymerase chain reaction
OKR	Optokinetic response
OMR	Optomotor response
SF	Spatial frequency
c/d	Cycles per degree
ERG	Electroretinography
μV	Microvolts
ms	Milliseconds
FITC	Fluorescein isothiocyanate
Con A	Concanavalin A
PBS	Phosphate-buffered saline
FBS	Fetal bovine serum
PFA	Paraformaldehyde
PBS-T	Phosphate-buffered saline containing 0.1% Triton X-100
BRN3A	Brain-specific homeobox/POU domain protein 3A
RBPMS	RNA-Binding Protein with Multiple Splicing
3-NT	3-nitrotyrosine
4-HNE	4-hydroxynonenal
OCT	Optimal cutting temperature
MPO	Myeloperoxidase
γH2AX	H2A histone family member X
DAPI	4′,6-diamidino-2-phenylindole
RIPA	Radioimmunoprecipitation assay
SDS–PAGE	Sodium dodecyl sulfate–polyacrylamide gel electrophoresis
ELISA	Enzyme-linked immunosorbent assay
TNFα	Tumor necrosis factor α
IL-10	Interleukin 10
IL-1β	Interleukin 1 beta
IL-6	Interleukin 6
SEM	Standard Error of the Mean
GCL	Ganglion cell layer
INL	Inner nuclear layer
BRB	Blood–retinal barrier
ICAM-1	Intercellular adhesion molecule-1
