# Supplementary material for: Vascular Deletion of HDAC6 Ameliorates Diabetic Retinal Microangiopathy and Diabetic Retinopathy in an Experimental Model of Type 1 Diabetes
Source: Cells. 2026 Jul 10;15(14):1244. doi: 10.3390/cells15141244 (PMC13406370; doi:10.3390/cells15141244)
Supplement: Supplementary file 1 [file cells-15-01244-s001.zip › cells-4342199-supplementary.pdf]

Supplementary Materials

Supplementary Table S1

Metabolic Parameters: **Two-Way ANOVA with Tukey Post-Hoc Test**

Parameter	Timepoint	HDAC6 flox/flox Mean ± SEM	DB HDAC6 flox/flox Mean ± SEM	DB HDAC6 cre flox flox Mean ± SEM	ANOVA p-value	HDAC6 ff vs DB HDAC6 ff	HDAC6 ff vs DB HDAC6 cre ff	DB HDAC6 ff vs DB HDAC6 cre ff	Significance
**Body weight (g)**	12 WEEKS	23.42 ± 0.60	22.65 ± 0.51	22.60 ± 0.28	0.4177	0.5074	0.4646	0.9971	ns
	18 WEEKS	28.20 ± 0.92	22.30 ± 0.54	22.89 ± 0.47	**<0.0001**	**<0.0001**	**<0.0001**	0.8091	***
	24 WEEKS	32.65 ± 0.67	18.84 ± 0.57	19.20 ± 0.45	**<0.0001**	**<0.0001**	**<0.0001**	0.8972	***
**Blood Glucose (mg/dl)**	12 WEEKS	120.40 ± 5.80	495.60 ± 25.37	464.30 ± 28.48	**<0.0001**	**<0.0001**	**<0.0001**	0.5871	***
	18 WEEKS	149.50 ± 10.06	522.40 ± 17.81	460.60 ± 31.24	**<0.0001**	**<0.0001**	**<0.0001**	0.1250	***
	24 WEEKS	141.60 ± 10.33	562.30 ± 10.90	521.90 ± 20.41	**<0.0001**	**<0.0001**	**<0.0001**	0.1436	***
**HbA1c (mmol/mol)**	12 WEEKS	4.01 ± 0.10	8.65 ± 0.44	7.68 ± 0.54	**<0.0001**	**<0.0001**	**<0.0001**	0.2284	***
	18 WEEKS	3.93 ± 0.09	8.88 ± 0.38	8.27 ± 0.52	**<0.0001**	**<0.0001**	**<0.0001**	0.4969	***
	24 WEEKS	4.09 ± 0.07	9.14 ± 0.39	8.38 ± 0.49	**<0.0001**	**<0.0001**	**<0.0001**	0.3203	***

*Legend: *** p<0.001 | ** p<0.01 | * p<0.05 | ns = not significant | Yellow = p<0.05 (Tukey post-hoc) | Values shown as Mean ± SEM*
